# Supplementary material for: Development of a 3D tracking system for multiple marmosets under free-moving conditions
Source: Commun Biol. 2024 Feb 21;7:216. doi: 10.1038/s42003-024-05864-9 (PMC10881507; doi:10.1038/s42003-024-05864-9)
Supplement: Supplementary file 2 — Supplementary Information [file 42003_2024_5864_MOESM2_ESM.pdf]

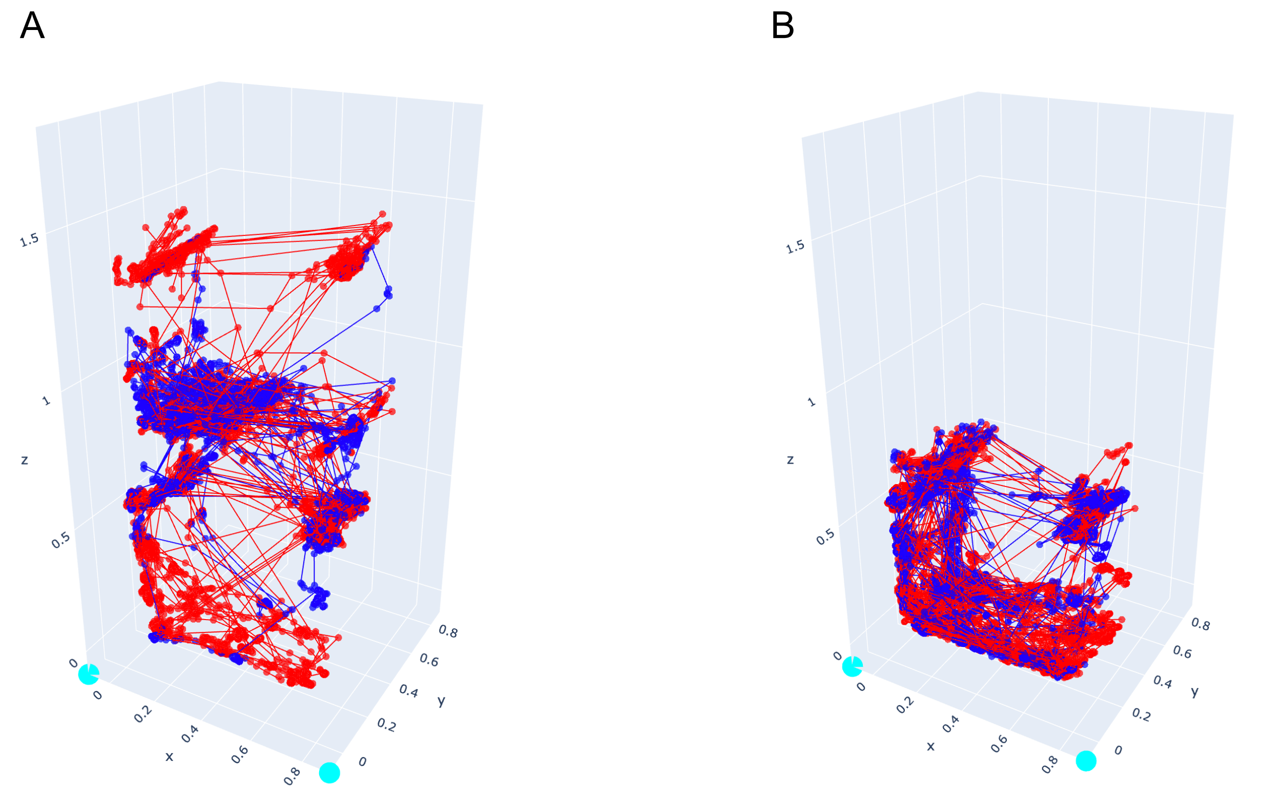

Supplementary Fig. 1. Tracking trajectory when changing cage space in preliminary study
Tracking trajectories when cage space is reduced using cage partitions. (A) Whole cage (B) Half cage. Blue: male marmoset, red: female marmoset.

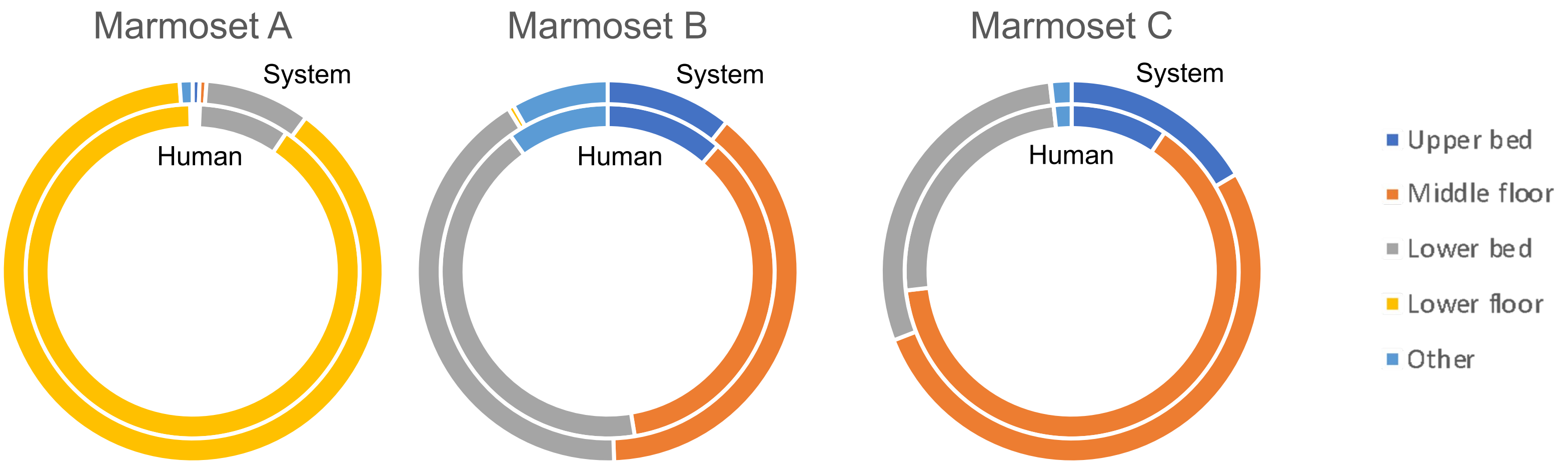

Supplementary Fig. 2. Comparison of visual observations and system data for time spent (per hour) by each marmoset.

The outside circle represents the result from the system, and the inside circle represents the result from human visual inspection. The results are shown as a percentage of time spent at each location.

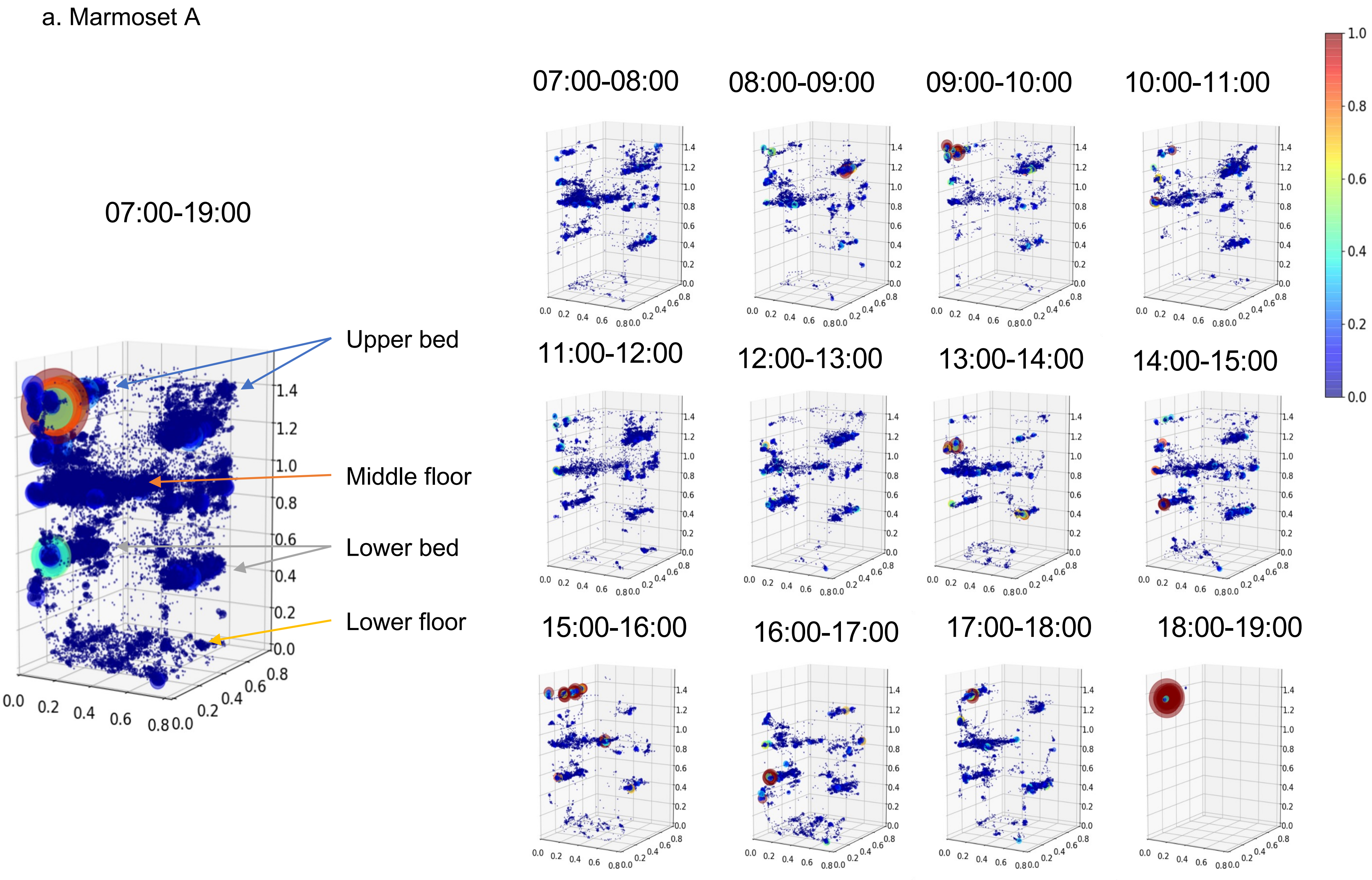
a. Marmoset A
07:00-19:00
Upper bed
Middle floor
Lower bed
Lower floor
07:00-08:00
08:00-09:00
09:00-10:00
10:00-11:00
11:00-12:00
12:00-13:00
13:00-14:00
14:00-15:00
15:00-16:00
16:00-17:00
17:00-18:00
18:00-19:00
0.0
0.2
0.4
0.6
0.8
1.0

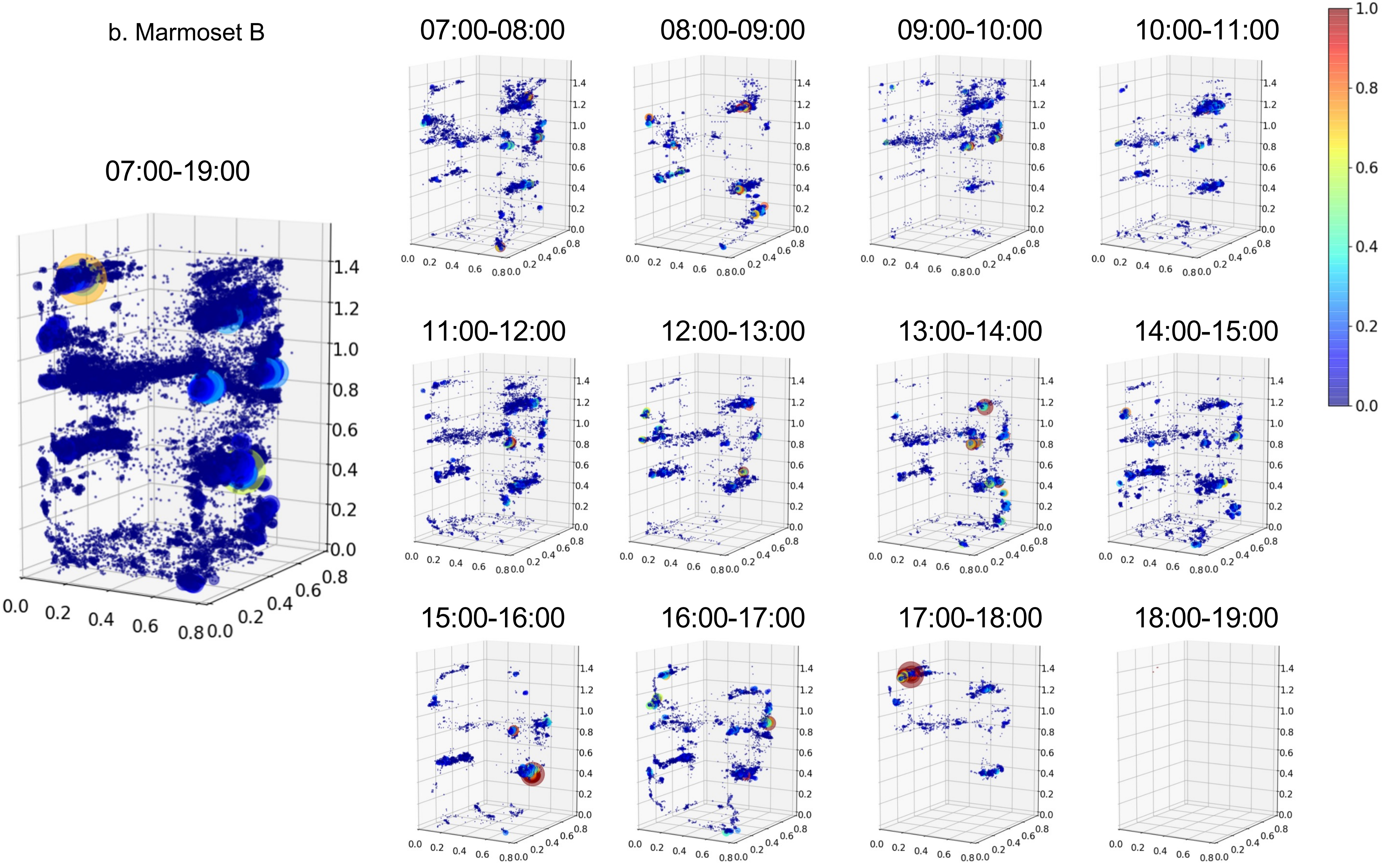

b. Marmoset B
07:00-19:00
07:00-08:00
08:00-09:00
09:00-10:00
10:00-11:00
11:00-12:00
12:00-13:00
13:00-14:00
14:00-15:00
15:00-16:00
16:00-17:00
17:00-18:00
18:00-19:00
0.0
0.2
0.4
0.6
0.8
1.0
1.2
1.4

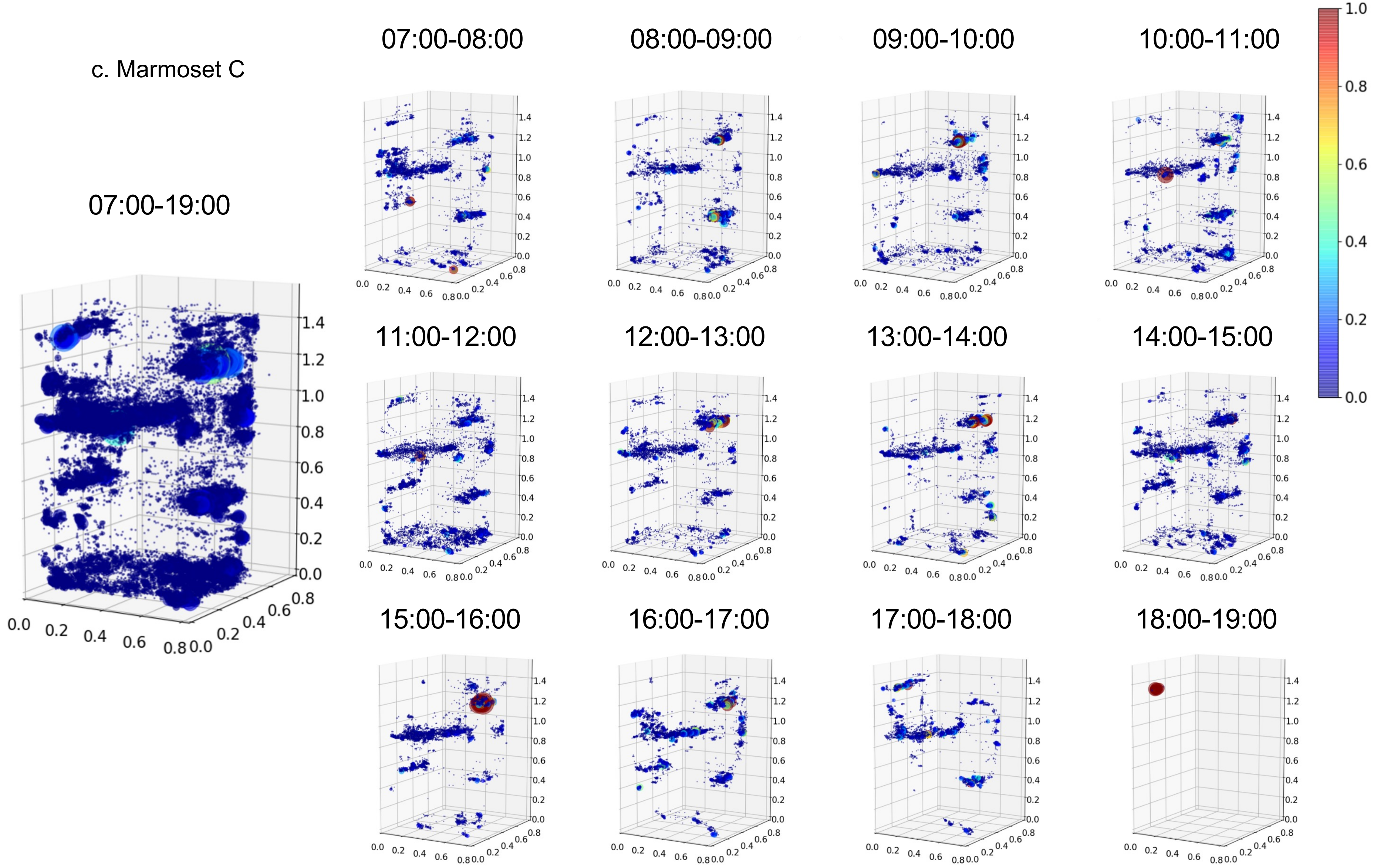

Supplementary Fig. 3. Place preference of marmosets for 1 day.
Comparison of the time spent by marmosets in the cage for 1 day. Each larger left graph shows activity during the whole day. The graphs on the right show hourly activity. Locations where marmosets spent more than 1% of 1 day are shown in red, and others are shown as color bars. (a) Marmoset A, (b) Marmoset B, and (c) Marmoset C.

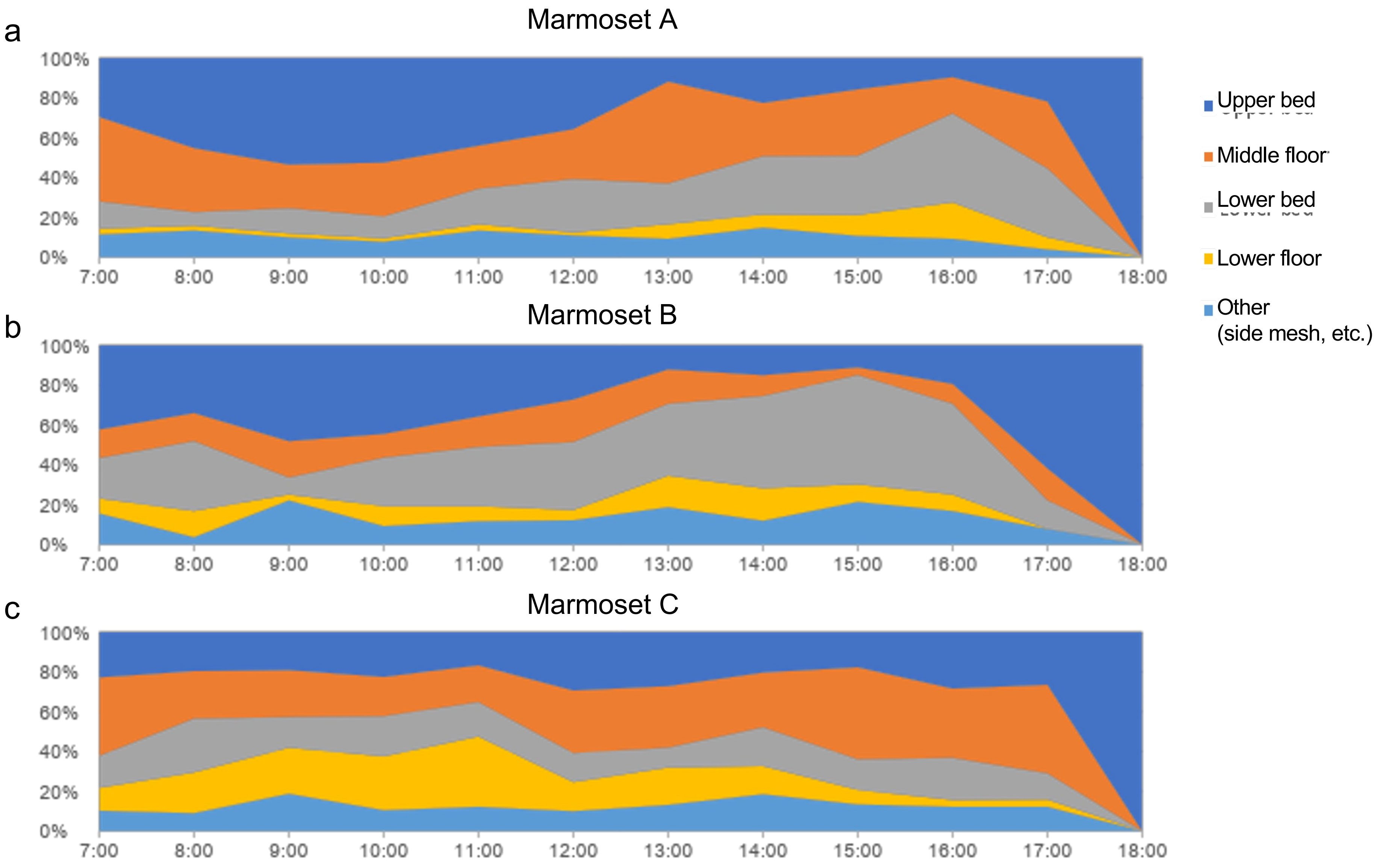

Supplementary Fig. 4. Variation in time spent per hour at each location.
The rate of stay at each measurement point was analyzed hourly. The four measurement points were the upper bed, middle floor, lower bed, and lower floor. (a) Marmoset A, (b) Marmoset B, and (c) Marmoset C.

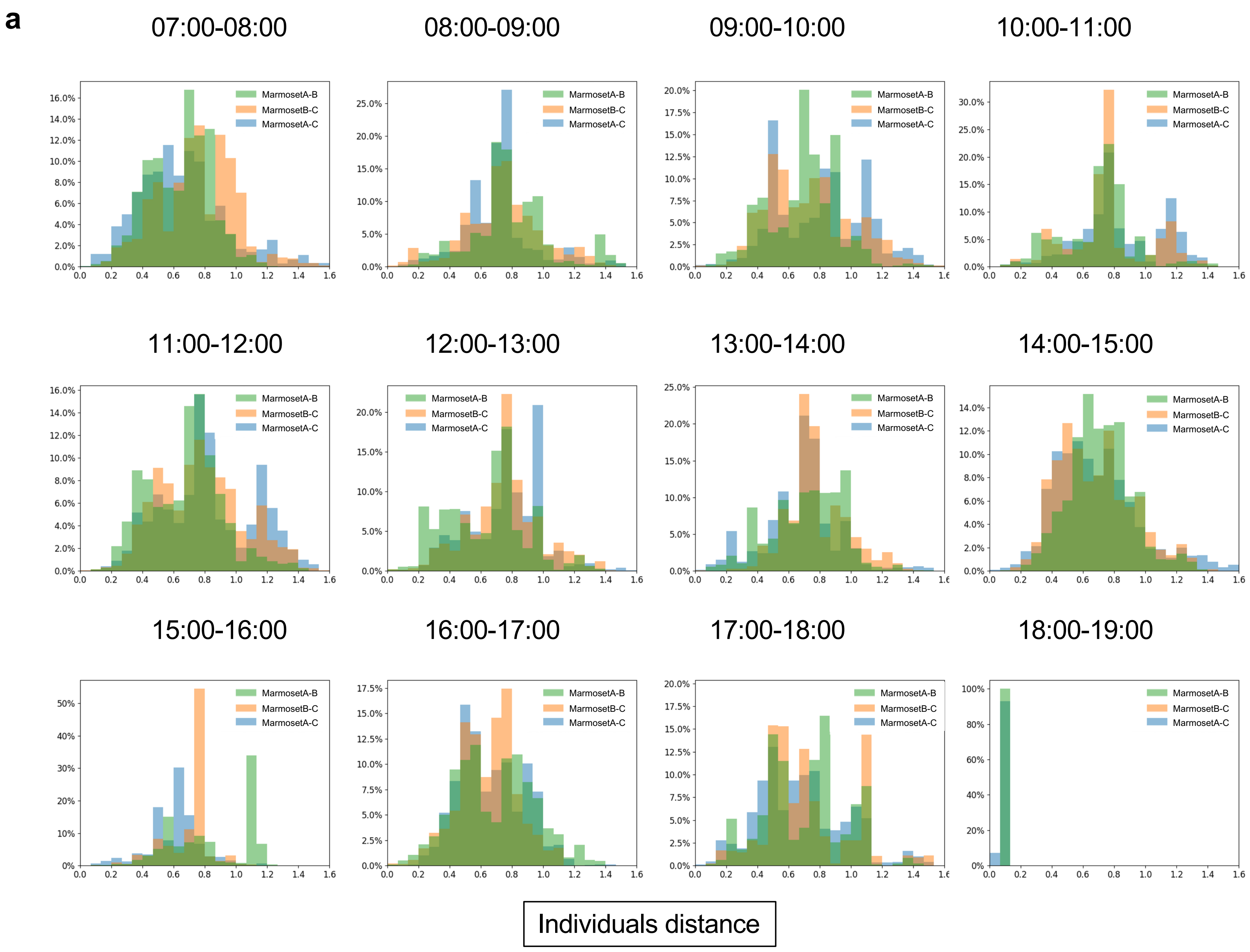
a
07:00-08:00
08:00-09:00
09:00-10:00
10:00-11:00
11:00-12:00
12:00-13:00
13:00-14:00
14:00-15:00
15:00-16:00
16:00-17:00
17:00-18:00
18:00-19:00
MarmosetA-B
MarmosetB-C
MarmosetA-C
Individuals distance

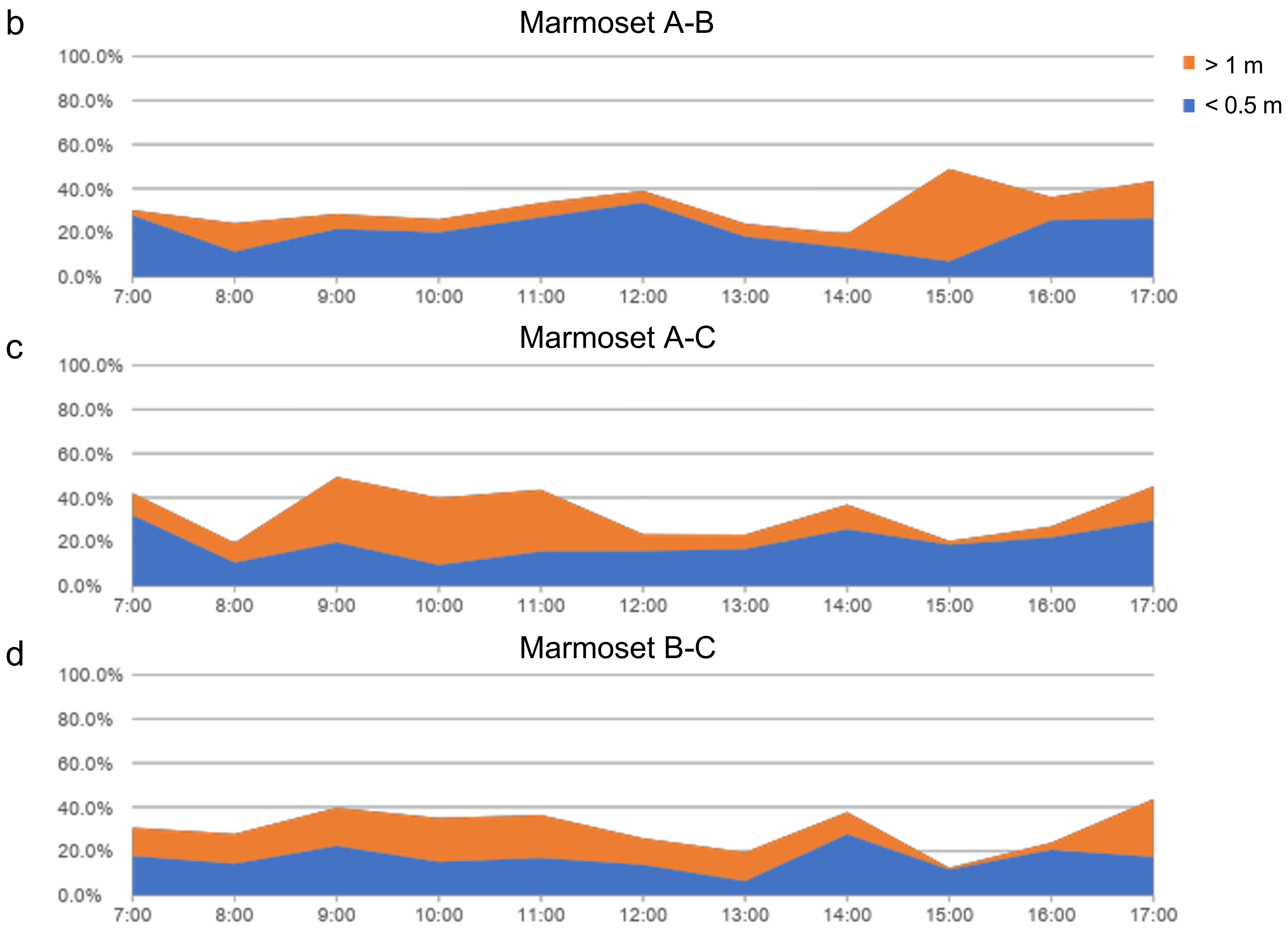

Supplementary Fig. 5. Time variation of inter-individual distance.
(a) Histograms of inter-individual distances for each individual pair at each time. (b-c)The rate of close and far time are extracted, with blue indicating when the distance was less than 0.5 m and orange when the distance was greater than 1 m. (b) Marmoset A-Marmoset B (c) Marmoset A-Marmoset C, and (d) Marmoset B-Marmoset C.

a

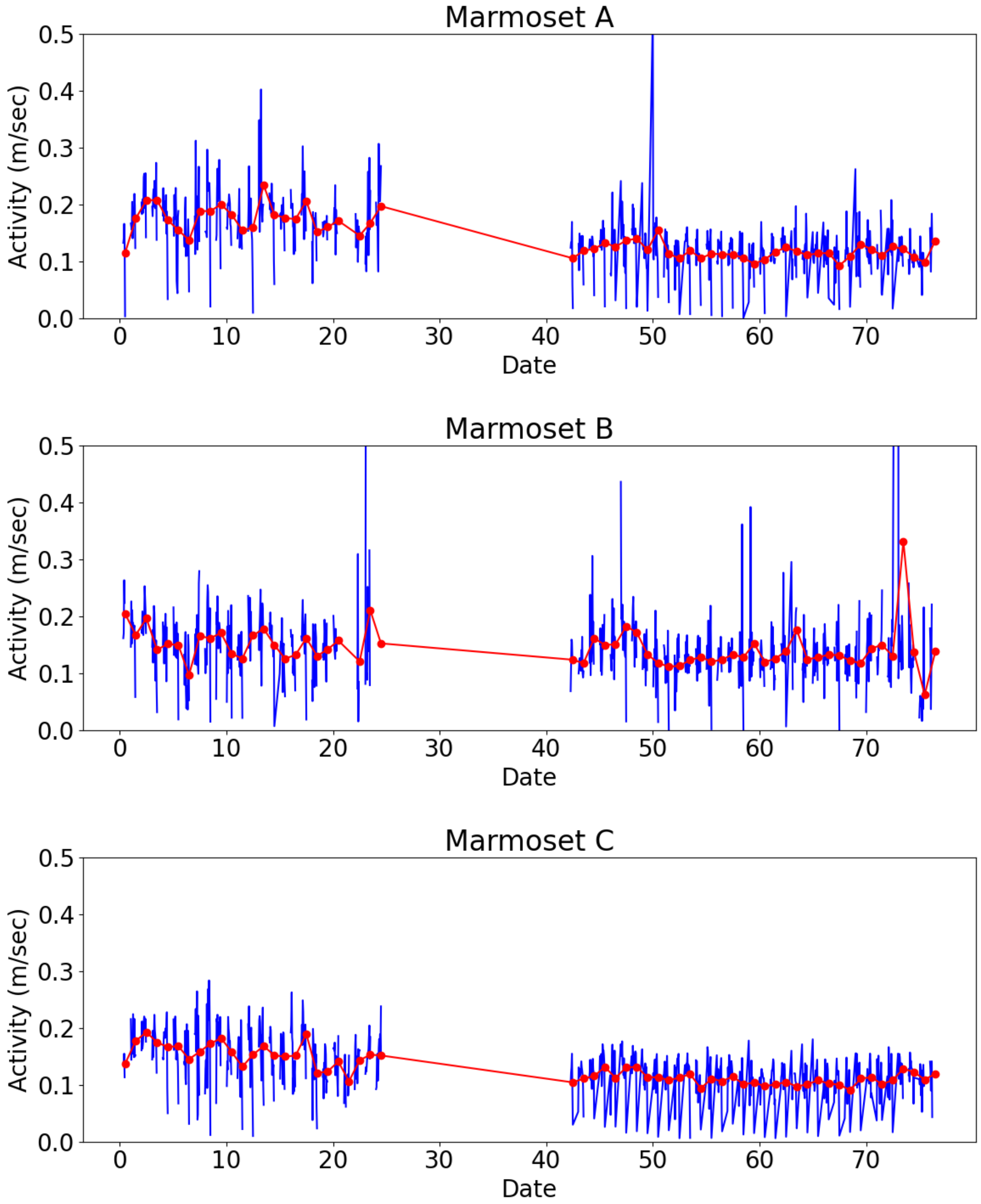

Marmoset A
Activity (m/sec)
0.0
0.1
0.2
0.3
0.4
0.5
0
10
20
30
40
50
60
70
Date
Marmoset B
Activity (m/sec)
0.0
0.1
0.2
0.3
0.4
0.5
0
10
20
30
40
50
60
70
Date
Marmoset C
Activity (m/sec)
0.0
0.1
0.2
0.3
0.4
0.5
0
10
20
30
40
50
60
70
Date

b

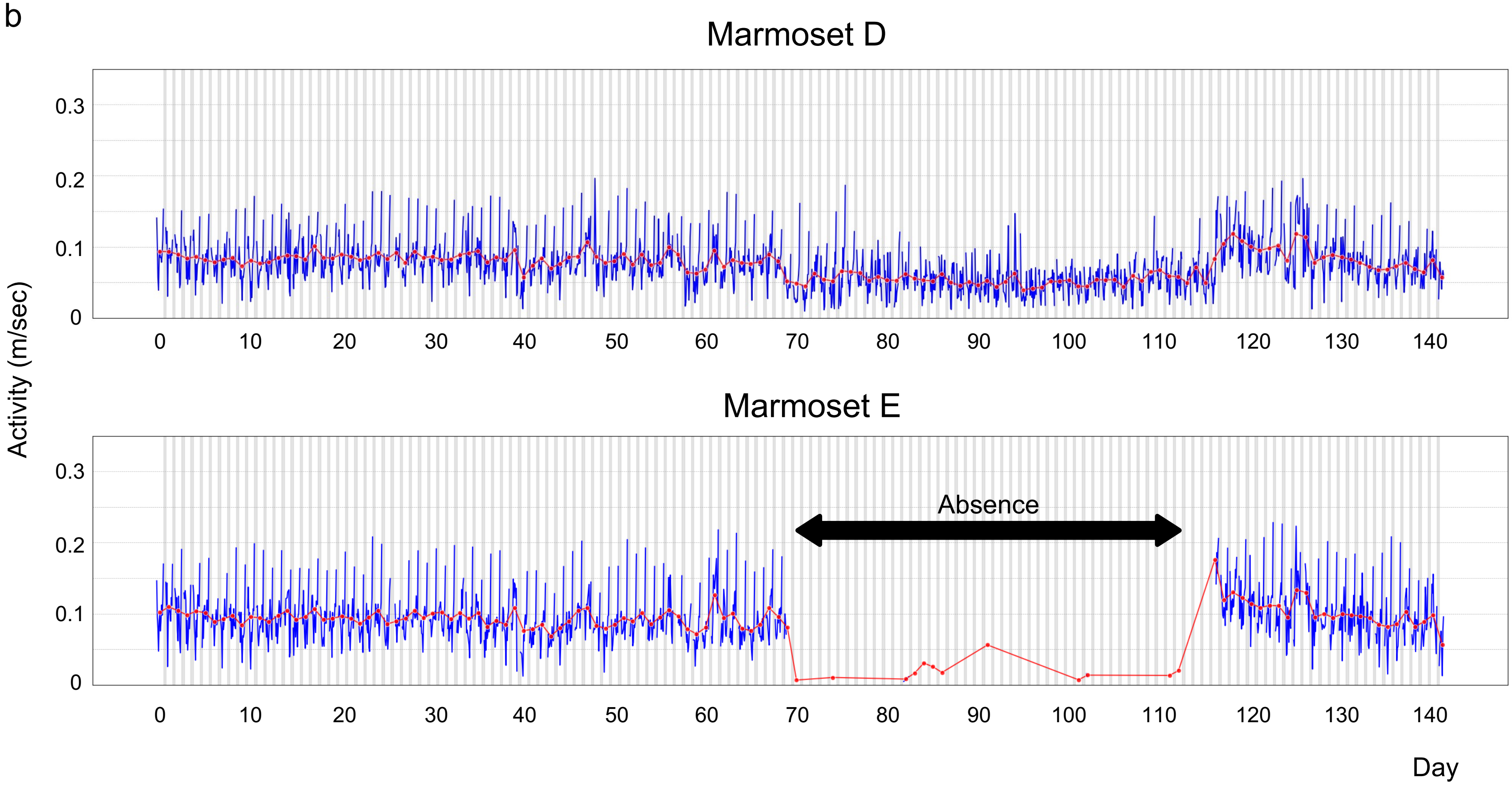

Supplementary Fig. 6. Activity amount for several months
The activity amount for each marmoset obtained by running the system for several months. The vertical axis shows the average moving distance per second, the blue line shows the hourly activity amount, and the red line shows the daily average value. (a) The activity of marmosets A, B, and C was investigated for about two months. The system was down for maintenance from 25 to 41 days. (b) The activity of marmosets D (2 years old, male) and E (2 years old, female) was investigated in another cage for about four months. Marmoset E was absent for other experiments from 69 to 115 days, so data are missing.

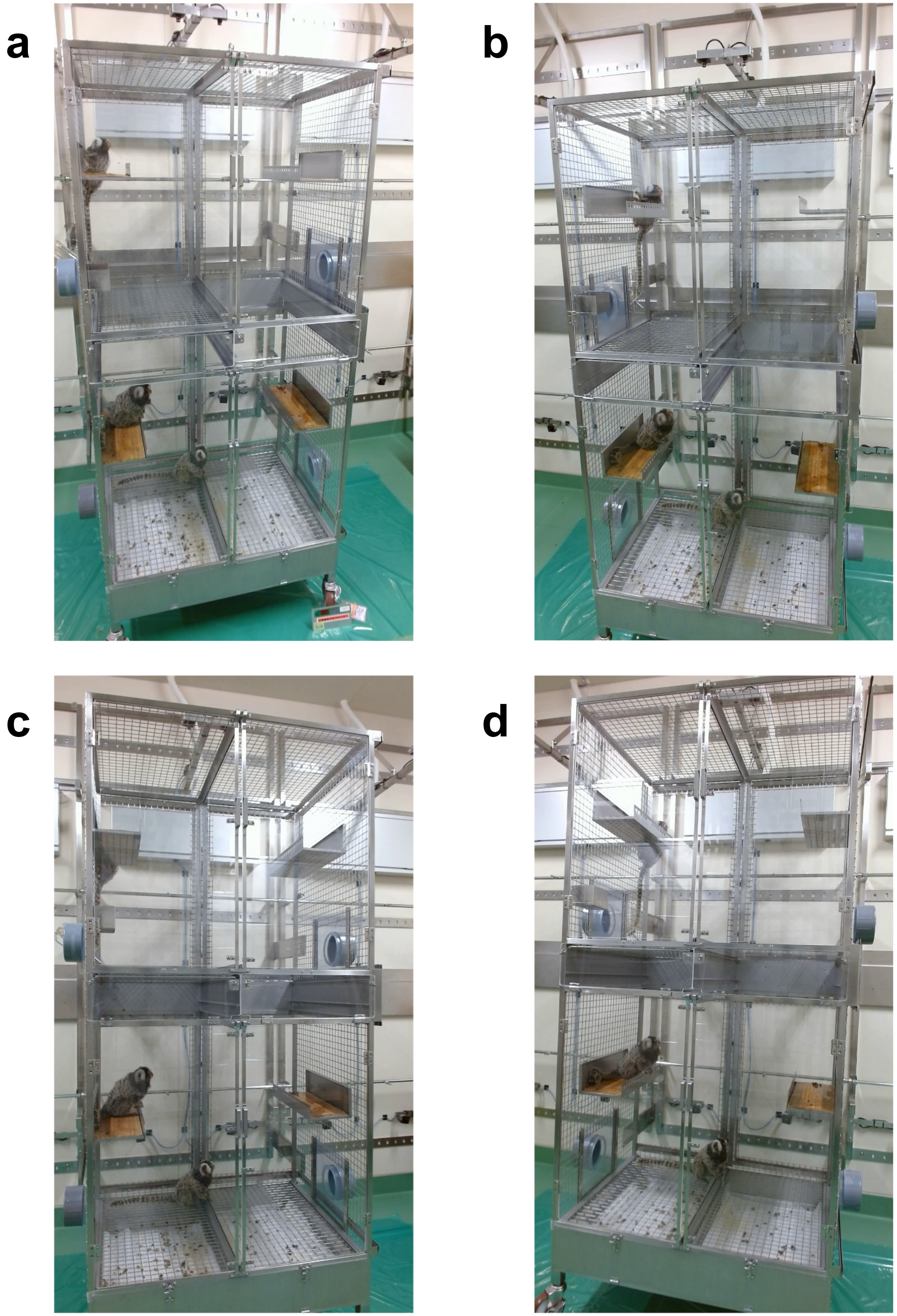

Supplementary Fig. 7. Photographs taken using four cameras.
(a) Top left, (b) Top right, (c) Bottom left, (d) Bottom right
