# Supplementary material for: Development of a 3D tracking system for multiple marmosets under free-moving conditions
Source: Commun Biol. 2024 Feb 21;7:216. doi: 10.1038/s42003-024-05864-9 (PMC10881507; doi:10.1038/s42003-024-05864-9)
Supplement: Supplementary file 3 — Description of additional supplementary files [file 42003_2024_5864_MOESM3_ESM.docx]

Description of Additional Supplementary Files

**File name:** Supplementary Mov. 1

**Description:** Video of 3D tracking of multi-headed marmosets using Lidar and video tracking

**File name:** Supplementary Mov. 2

**Description:** Video of a marmoset jumping and tracking when it is moving quickly

**File name:** Supplementary Mov. 3

**Description:** Video of a marmoset tracking when it hides behind something

**File name:** Supplementary Mov. 4

**Description:** 3D tracking of marmosets attached with color markers by our tracking system

**File name**: Supplementary Mov. 5

**Description:** Tracking with some moving enrichments

**File name:** Supplementary Mov. 6

**Description:** 3D tracking in a grooming situation

**File name:** Supplementary Mov. 7

**Description:** Tracking when an animal exits off-screen

**File name:** Supplementary Mov. 8

**Description:** Automatic tracking of a marmoset moving at night

**File name:** Supplementary Data 1

**Description:** Code for face identification with VGG19

**File name:** Supplementary Data 2

**Description:** Code for grooming detection

**File name:** Supplementary Data 3

**Description:** Source data for Fig. 4, 5, 6
