# Supplementary material for: Development of a 3D tracking system for multiple marmosets under free-moving conditions
Source: Commun Biol. 2024 Feb 21;7:216. doi: 10.1038/s42003-024-05864-9 (PMC10881507; doi:10.1038/s42003-024-05864-9)
Supplement: Supplementary file 6 — Supplementary Mov. 3 [file 42003_2024_5864_MOESM6_ESM.pptx]

## Slide 1
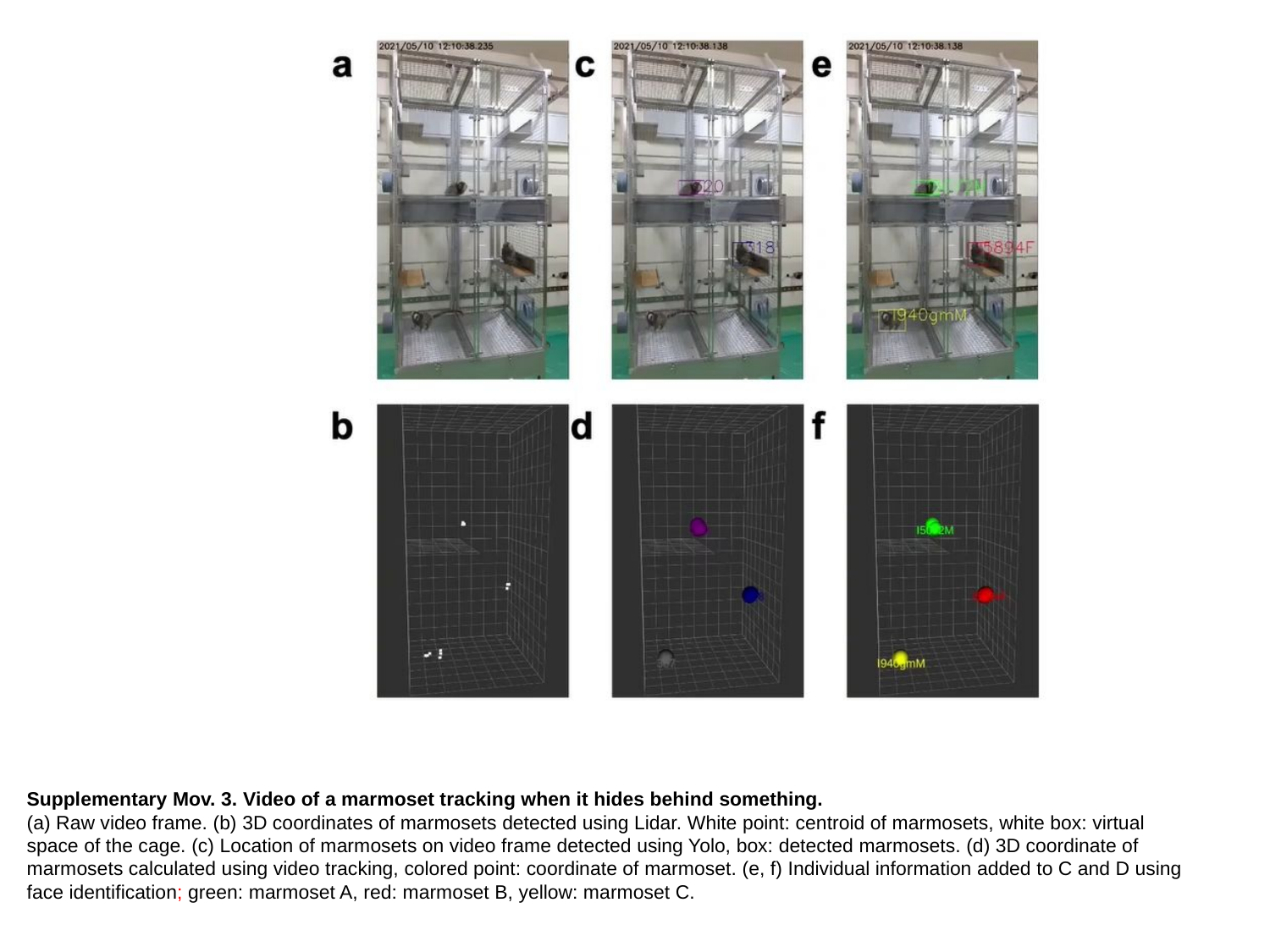

Supplementary Mov. 3. Video of a marmoset tracking when it hides behind something.
(a) Raw video frame. (b) 3D coordinates of marmosets detected using Lidar. White point: centroid of marmosets, white box: virtual space of the cage. (c) Location of marmosets on video frame detected using Yolo, box: detected marmosets. (d) 3D coordinate of marmosets calculated using video tracking, colored point: coordinate of marmoset. (e, f) Individual information added to C and D using face identification; green: marmoset A, red: marmoset B, yellow: marmoset C.
