# Supplementary material for: Development of a 3D tracking system for multiple marmosets under free-moving conditions
Source: Commun Biol. 2024 Feb 21;7:216. doi: 10.1038/s42003-024-05864-9 (PMC10881507; doi:10.1038/s42003-024-05864-9)
Supplement: Supplementary file 7 — Supplementary Mov. 4 [file 42003_2024_5864_MOESM7_ESM.pptx]

## Slide 1
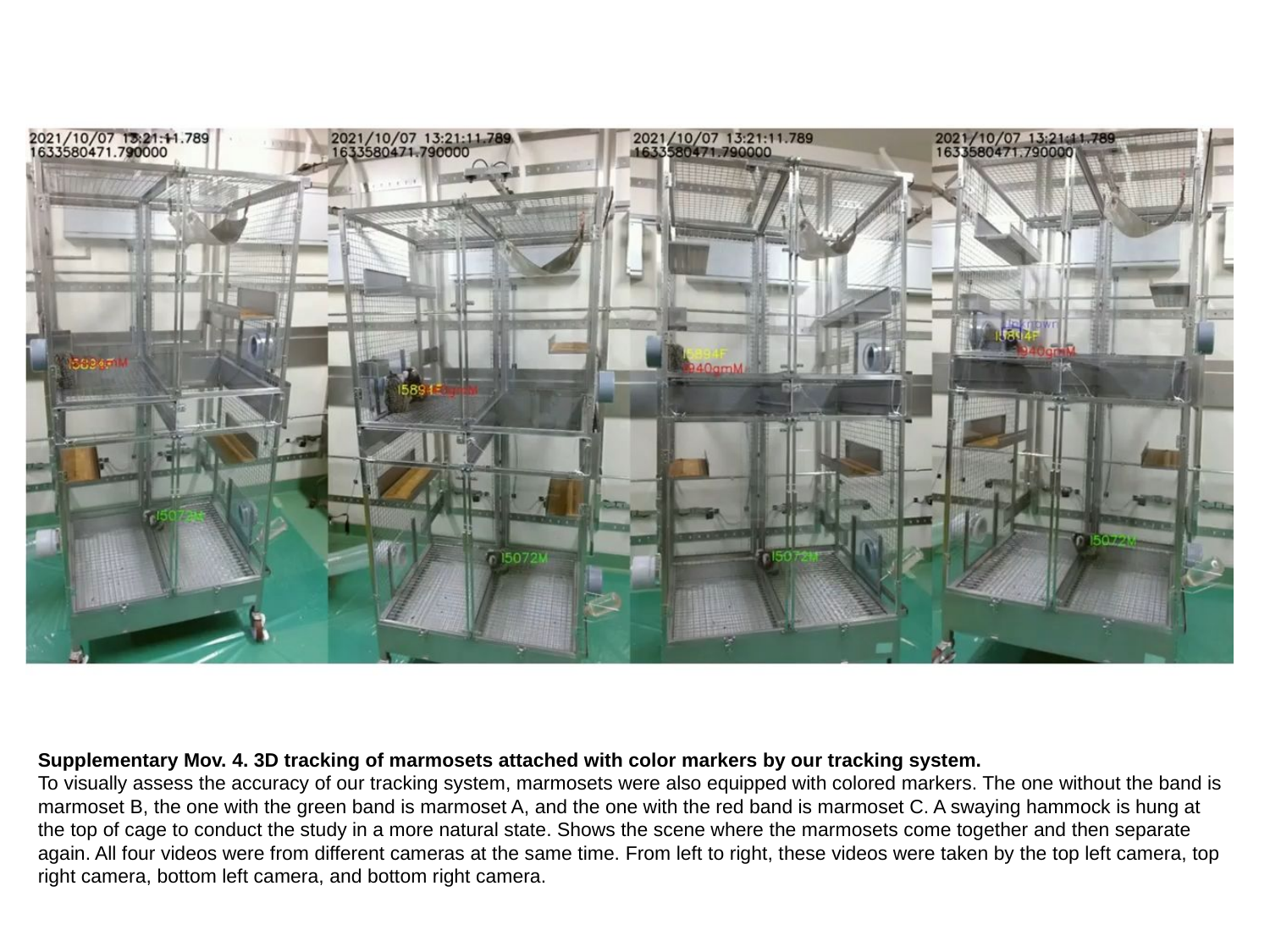

Supplementary Mov. 4. 3D tracking of marmosets attached with color markers by our tracking system.
To visually assess the accuracy of our tracking system, marmosets were also equipped with colored markers. The one without the band is marmoset B, the one with the green band is marmoset A, and the one with the red band is marmoset C. A swaying hammock is hung at the top of cage to conduct the study in a more natural state. Shows the scene where the marmosets come together and then separate again. All four videos were from different cameras at the same time. From left to right, these videos were taken by the top left camera, top right camera, bottom left camera, and bottom right camera.
