# Supplementary material for: Development of a 3D tracking system for multiple marmosets under free-moving conditions
Source: Commun Biol. 2024 Feb 21;7:216. doi: 10.1038/s42003-024-05864-9 (PMC10881507; doi:10.1038/s42003-024-05864-9)
Supplement: Supplementary file 8 — Supplementary Mov. 5 [file 42003_2024_5864_MOESM8_ESM.pptx]

## Slide 1
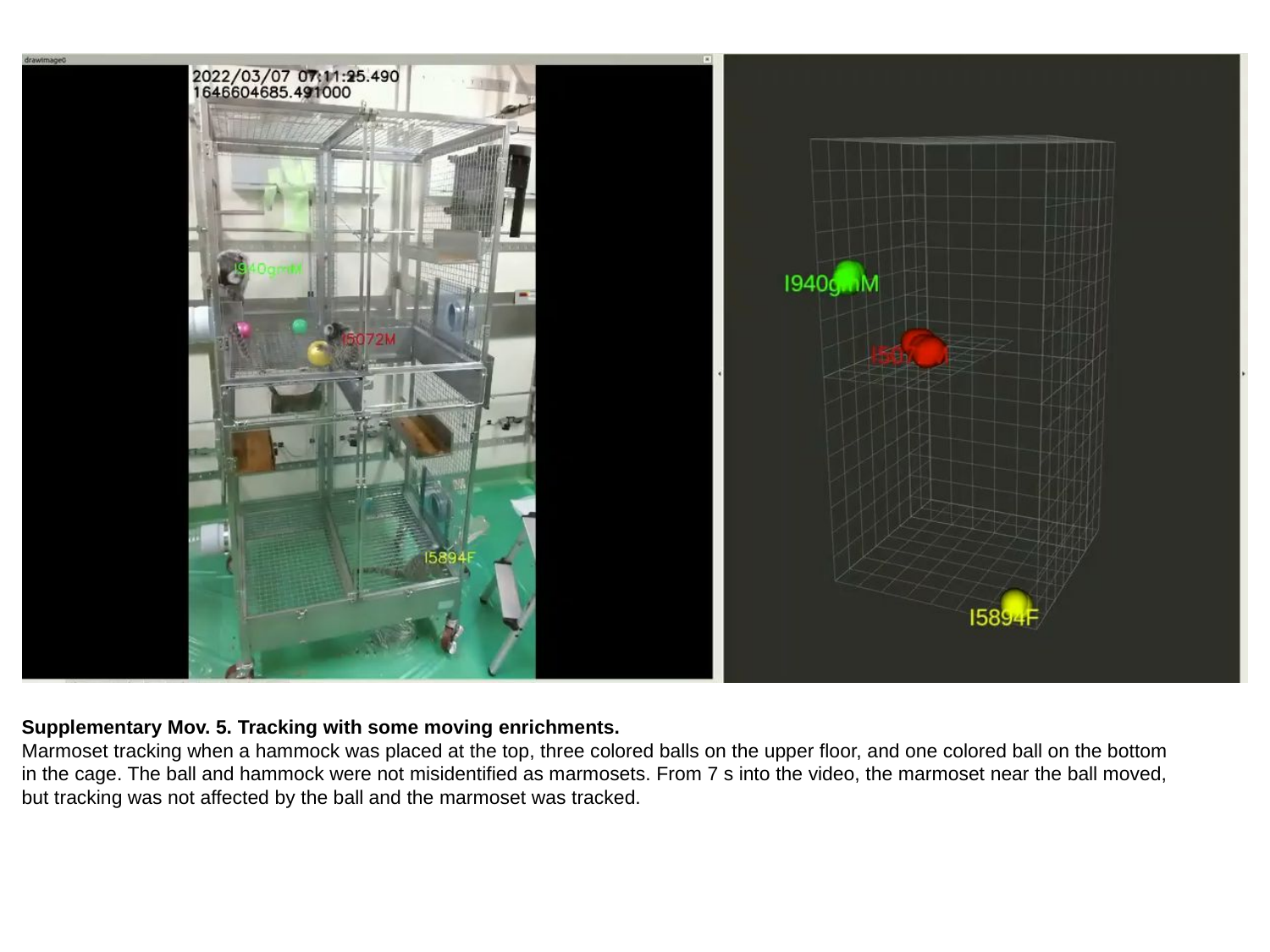

Supplementary Mov. 5. Tracking with some moving enrichments.
Marmoset tracking when a hammock was placed at the top, three colored balls on the upper floor, and one colored ball on the bottom in the cage. The ball and hammock were not misidentified as marmosets. From 7 s into the video, the marmoset near the ball moved, but tracking was not affected by the ball and the marmoset was tracked.
