# Supplementary material for: Development of a 3D tracking system for multiple marmosets under free-moving conditions
Source: Commun Biol. 2024 Feb 21;7:216. doi: 10.1038/s42003-024-05864-9 (PMC10881507; doi:10.1038/s42003-024-05864-9)
Supplement: Supplementary file 9 — Supplementary Mov. 6 [file 42003_2024_5864_MOESM9_ESM.pptx]

## Slide 1
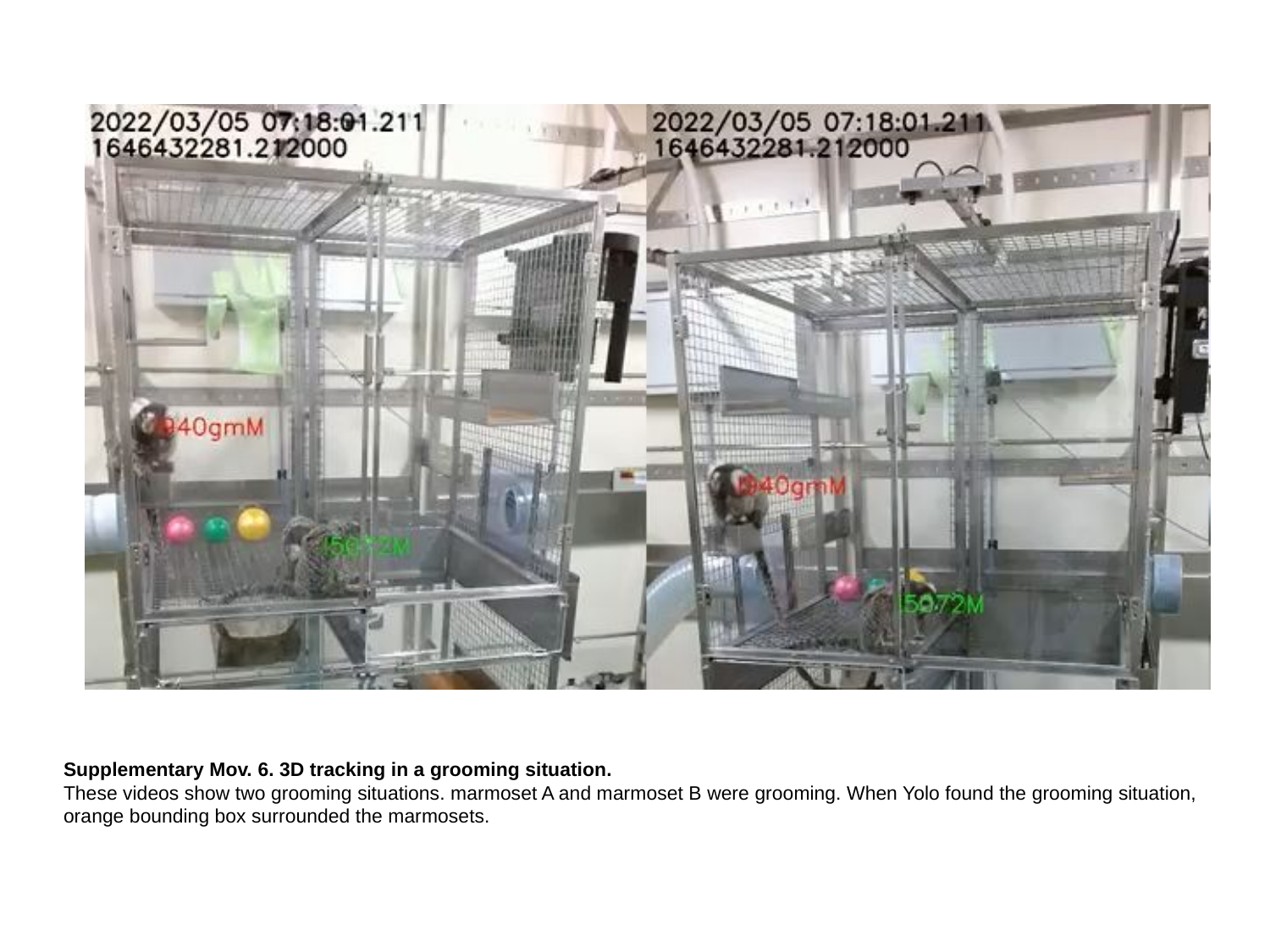

Supplementary Mov. 6. 3D tracking in a grooming situation.
These videos show two grooming situations. marmoset A and marmoset B were grooming. When Yolo found the grooming situation, orange bounding box surrounded the marmosets.
