# Supplementary material for: Development of a 3D tracking system for multiple marmosets under free-moving conditions
Source: Commun Biol. 2024 Feb 21;7:216. doi: 10.1038/s42003-024-05864-9 (PMC10881507; doi:10.1038/s42003-024-05864-9)
Supplement: Supplementary file 10 — Supplementary Mov. 7 [file 42003_2024_5864_MOESM10_ESM.pptx]

## Slide 1
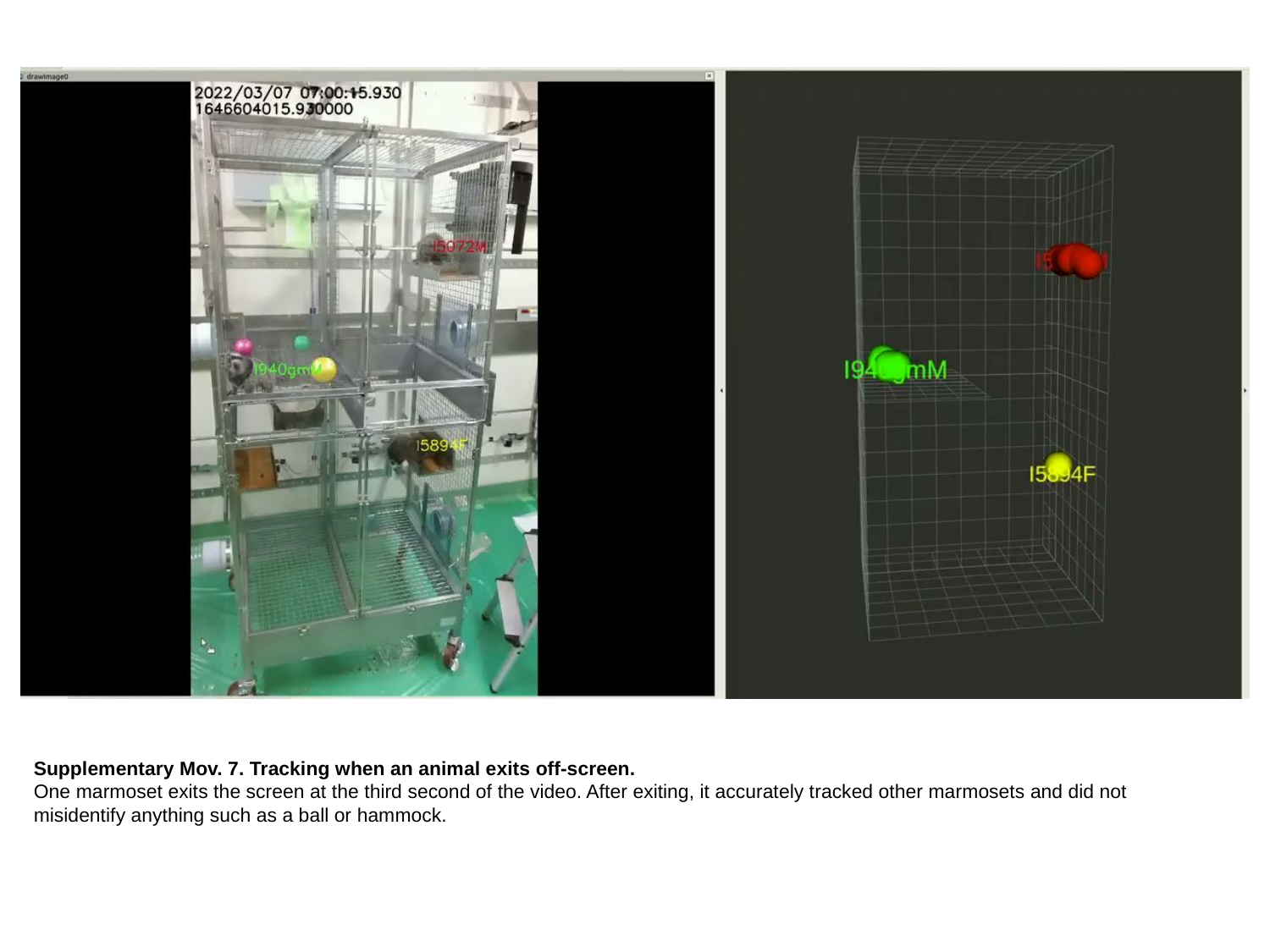

Supplementary Mov. 7. Tracking when an animal exits off-screen.
One marmoset exits the screen at the third second of the video. After exiting, it accurately tracked other marmosets and did not misidentify anything such as a ball or hammock.
