# Supplementary material for: Development of a 3D tracking system for multiple marmosets under free-moving conditions
Source: Commun Biol. 2024 Feb 21;7:216. doi: 10.1038/s42003-024-05864-9 (PMC10881507; doi:10.1038/s42003-024-05864-9)
Supplement: Supplementary file 11 — Supplementary Mov. 8 [file 42003_2024_5864_MOESM11_ESM.pptx]

## Slide 1
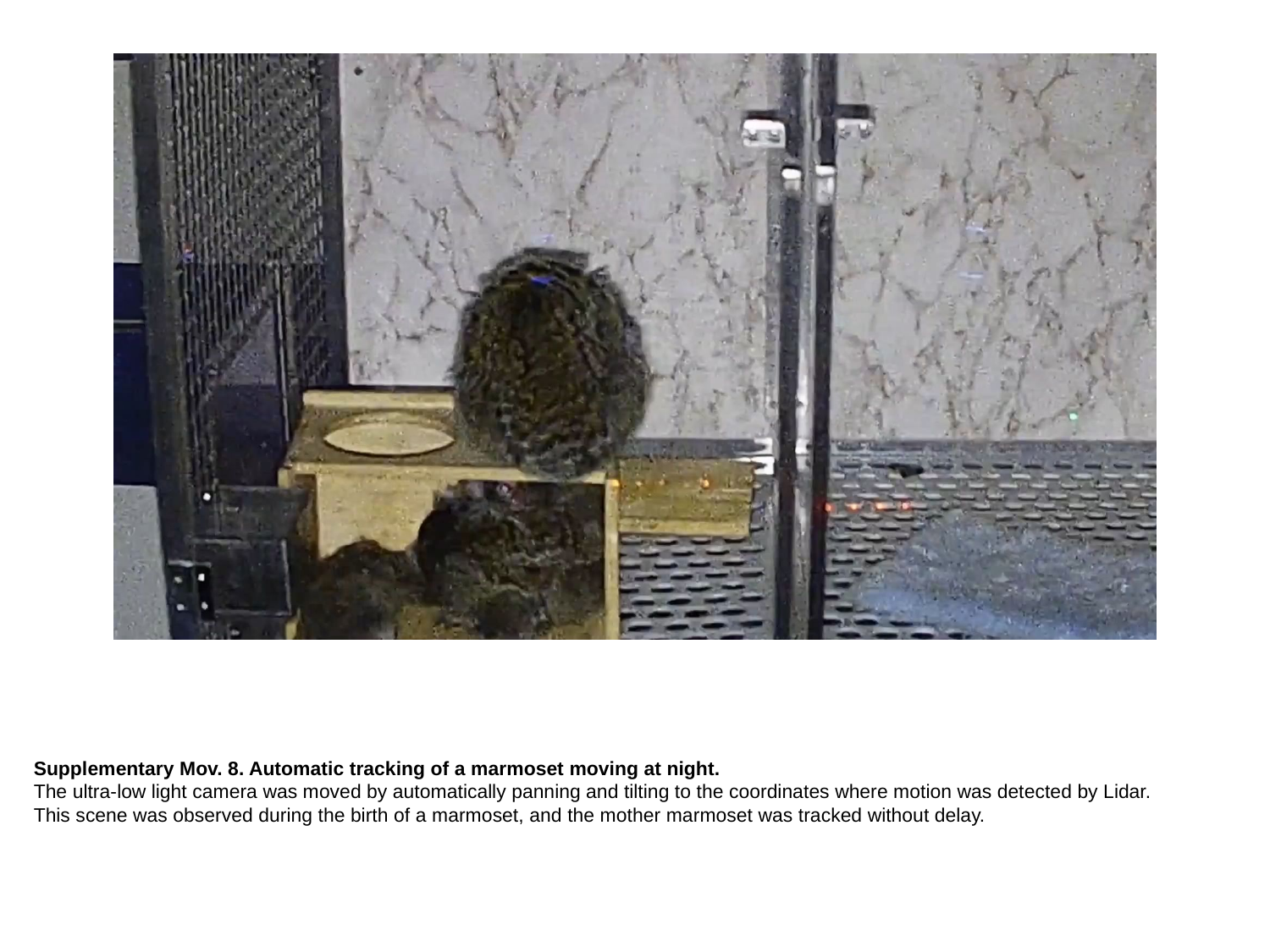

Supplementary Mov. 8. Automatic tracking of a marmoset moving at night.
The ultra-low light camera was moved by automatically panning and tilting to the coordinates where motion was detected by Lidar. This scene was observed during the birth of a marmoset, and the mother marmoset was tracked without delay.
