# Supplementary material for: Development of a 3D tracking system for multiple marmosets under free-moving conditions
Source: Commun Biol. 2024 Feb 21;7:216. doi: 10.1038/s42003-024-05864-9 (PMC10881507; doi:10.1038/s42003-024-05864-9)
Supplement: Supplementary file 12 — Supplementary Data 1 [file 42003_2024_5864_MOESM12_ESM.pdf]

Code for face identification with VGG19

```
import datetime
from tensorflow import keras
import numpy as np
import os
import sys
from tensorflow.keras import optimizers
from tensorflow.keras.callbacks import EarlyStopping
import glob
import pandas as pd
import tensorflow.keras
import tensorflow.compat.v1 as tf
from PIL import Image as PILImage
import cv2
import csv
from sklearn.metrics import confusion_matrix

os.environ["TF_FORCE_GPU_ALLOW_GROWTH"]= "true"



def train_face(setting_layer, setting_rate):
	print('****train_face****')

	#Path Settings
	# train_folder_path = 
'/home/behavior/Desktop/python/LearnFace/face_image/202109/learning'

	write_txt_path = os.path.join(result_folder_path, 'output_class_name_'+
d_today.strftime('%m%d%H%M')+'.txt')
	weight_save_path = os.path.join(result_folder_path,
'Marmoset_face_recognition_'+d_today.strftime('%m%d%H%M')+'_vgg19.hdf5')
	CLASS_NAME = os.listdir(train_folder_path)
	CLASS_NAME.sort()
	with open(write_txt_path, 'w') as f_txt:
```

```
        # f_txt.write(', '.join(CLASS_NAME))
        f_txt.write(str(CLASS_NAME))

    #Counting the number of training data and obtaining methods for padding training data
    data = str(len(glob.glob(train_folder_path+'/*/*.png')))
    image_padding = train_folder_path.split('/')[-1]

    #Model settings
    model_name = 'vgg19'
    IMAGE_SIZE = 224
    BATCH_SIZE = 32
    fixed_layer = setting_layer
    epoch = 50
    learning_rate = setting_rate
    momentum = 0.9
    os.environ["CUDA_VISIBLE_DEVICES"] = "0"

    from tensorflow.python.client import device_lib
    device_lib.list_local_devices()

    # Read VGG19 model without after FC layer
    base_model = keras.applications.vgg19.VGG19(weights='imagenet', include_top=False,
input_shape=(224, 224,3))
    x = base_model.output
    x = keras.layers.GlobalAveragePooling2D()(x)
    x = keras.layers.Dense(1024, activation='relu')(x)
    predictions = keras.layers.Dense(len(CLASS_NAME), activation='softmax')(x)
    model = keras.models.Model(inputs=base_model.input, outputs=predictions)

    # Fixed layer
    for layer_no in range(fixed_layer):
        layer = model.layers[layer_no]
        layer.trainable = False

    # Optimizer
    optimizer = keras.optimizers.SGD(lr=learning_rate, momentum=momentum)
```

```
    # optimizer = keras.optimizers.Adagrad(lr=learning_rate, epsilon=None, decay=0.0)
    # optimizer = keras.optimizers.Adam(lr=learning_rate, beta_1=0.9, beta_2=0.999,
epsilon=1e-08)
    model.compile(optimizer=optimizer,
        loss='categorical_crossentropy',
        metrics=['accuracy'])

    model.summary()

    train_datagen = keras.preprocessing.image.ImageDataGenerator(
        rescale = 1.0 / 255.0,
        validation_split=0.2)

    test_datagen = keras.preprocessing.image.ImageDataGenerator(
        rescale = 1.0 / 255.0,
        )

    train_generator = train_datagen.flow_from_directory(
        train_folder_path,
        target_size = (IMAGE_SIZE, IMAGE_SIZE),
        batch_size = BATCH_SIZE,
            classes = CLASS_NAME,
        seed = 1121,
        class_mode = 'categorical',
        shuffle = True
        )

    validation_generator = train_datagen.flow_from_directory(
        train_folder_path,
        target_size = (IMAGE_SIZE, IMAGE_SIZE),
        batch_size = BATCH_SIZE,
            classes = CLASS_NAME,
        seed = 1121,
        class_mode = 'categorical',
        shuffle = False
        )
```

```
    # EaelyStopping setting
    early_stopping =    EarlyStopping(
                            monitor='val_loss',
                            # min_delta=0,
                            min_delta=1e-5,
                            patience=2,
                            mode='auto')

    hist = model.fit_generator(
        train_generator,
        epochs = epoch,
        verbose = 1,
        validation_data = validation_generator,
        workers = 4,
        callbacks=[early_stopping]
        )

    model.save(weight_save_path)

    def model_config():
        dict = {'model':model_name, 'weight_number':d_today.strftime('%m%d%H%M'),
'learning_rate':str(learning_rate),
        'epoch':str(epoch),    'number_of_data':data,    'image_padding':image_padding,
'fixed_layer':str(fixed_layer)}
        df = pd.DataFrame(dict,index=['0'])
        df.to_csv(result_folder_path+'/model_config.csv')

    model_config()

def test_face():

    CLASS_NAME = os.listdir(train_folder_path)
    CLASS_NAME.sort()

    # setting
```

```
IMAGE_SIZE = 224
#CLASS_NAME = ['Unknown', 'I5072M', 'I5894F', 'I940gmM']
os.environ["CUDA_VISIBLE_DEVICES"] = "0"

from tensorflow.python.client import device_lib
device_lib.list_local_devices()


# Read VGG19 model without after FC layer
base_model = tensorflow.keras.applications.vgg19.VGG19(weights='imagenet',
include_top=False)
x = base_model.output
x = tensorflow.keras.layers.GlobalAveragePooling2D()(x)
x = tensorflow.keras.layers.Dense(1024, activation='relu')(x)
predictions = tensorflow.keras.layers.Dense(len(CLASS_NAME),
activation='softmax')(x)
model = tensorflow.keras.models.Model(inputs=base_model.input,
outputs=predictions)
model.load_weights(weight_path)
graph = tf.get_default_graph()

print('****test_face****')
name_list = ['I5072M', 'I5894F', 'I940gmM','Unknown']
for i in name_list:
    id_number = i


    # csv setting
    csv_file_name = id_number + 'face_recognition_result' + weight_number+ '.csv'
    csv_file_path = os.path.join(result_folder_path, csv_file_name)
    csv_f = open(csv_file_path, 'w')
    csv_writer = csv.writer(csv_f)


    def cv2pil(image):
        ''' OpenCV -> PIL '''
```

```
            new_image = image.copy()
            new_image = cv2.cvtColor(new_image, cv2.COLOR_BGR2RGB)
            new_image = PILImage.fromarray(new_image)
            return new_image

        image_dir_path = test_img_folder_path + '/' + id_number
        globpath = os.path.join(image_dir_path, "*")
        image_folder_entry_list_path = glob.glob(globpath) #[a,b,c]
        image_folder_entry_list_path.sort()
        for image_path in image_folder_entry_list_path:
            face_cv2_image = cv2.imread(image_path)
            face_image = cv2pil(face_cv2_image)
            face_image = face_image.resize((IMAGE_SIZE, IMAGE_SIZE))
            face_tensor = tensorflow.keras.preprocessing.image.array_to_img(face_image)
            face_tensor = np.expand_dims(face_tensor, axis=0)
            face_tensor =
tensorflow.keras.applications.vgg19.preprocess_input(face_tensor)
            face_tensor = face_tensor/255
            # get face id
            preds = model.predict(face_tensor)

            probability = preds[0][np.argmax(preds[0])]
            probability = '{:.3f}'.format(probability)

            #Classification Results
            classification_result = CLASS_NAME[np.argmax(preds[0])]
            frame_name = image_path.split('/')[-1].split('.')[0]
            image_file_name = frame_name + '_' + classification_result + '_' +
str(probability) + '.png'
            image_write_path = os.path.join(result_folder_path, image_file_name)
            #csv
            csv_writer.writerow([frame_name, CLASS_NAME[np.argmax(preds[0])],
preds[0][np.argmax(preds[0])], id_number])

def result_csv():
    print('****result_csv****')
```

```
csv_path = result_folder_path + "/accurancy_sum1.csv"

df = pd.DataFrame(columns = [])
for i in glob.glob(result_folder_path + "/*face_recognition_result*"):
    tmp = pd.read_csv(i, header=None)
    df = pd.concat([df, tmp])

pred_list = df.iloc[:, 1].tolist()
true_list = df.iloc[:, 3].tolist()
cm = confusion_matrix(true_list, pred_list, labels=['I5894F', 'I940gmM', 'I5072M',
'Unknown'])
df = pd.DataFrame(cm)

def confusion_matrix_csv():
    pd.DataFrame(cm).to_csv(csv_path,
            index = False,
            header = ['I5894F', 'I940gmM', 'I5072M', 'Unknown'])



def syuukei():
    #ACC1
    total = int(df.iat[0,0] + df.iat[0,1] + df.iat[0,2] + df.iat[0,3])
    ACC1_I5894F = int(df.iat[0,0])/(total - int(df.iat[0,3]))
    ACC1_I940gmM = int(df.iat[1,1])/(total - int(df.iat[1,3]))
    ACC1_I5072M = int(df.iat[2,2])/(total - int(df.iat[2,3]))
    ACC1_average = (ACC1_I5894F + ACC1_I5072M + ACC1_I940gmM)/3

    #ACC2
    ACC2_I5894F = int(df.iat[0,0])/total
    ACC2_I940gmM = int(df.iat[1,1])/total
    ACC2_I5072M = int(df.iat[2,2])/total
    ACC2_Unknown = int(df.iat[3,3])/total
    ACC2_average = (ACC2_I5894F + ACC2_I940gmM +
ACC2_I5072M+ACC2_Unknown) / 4

    df_model = pd.read_csv(result_folder_path + "/model_config.csv")
```

```
            df_model2 = df_model.drop(df_model.columns[0], axis=1)

            df_syuukei = df_model2.assign(ACC1_I5894F = ACC1_I5894F, ACC1_I940gmM =
ACC1_I940gmM,   ACC1_I5072M = ACC1_I5072M, ACC1_average = ACC1_average,
            ACC2_I5894F = ACC2_I5894F, ACC2_I940gmM = ACC2_I940gmM,
ACC2_I5072M = ACC2_I5072M, ACC2_Unknown=ACC2_Unknown, ACC2_average =
ACC2_average)
            df_syuukei.to_csv(result_folder_path + "/result.csv", header = True, index = False)


df_syuukei.to_csv('/home/behavior/Desktop/python/LearnFace/4_FaceTest/total_result_2
0211203.csv', mode='a', header=False, index = False)

      confusion_matrix_csv()
      syuukei()



#main
layer_list = [9, 12]
learning_rate_list = [1e-3]
train_data_list = ['r',
# 'rhs', 'rz',
'rzhs']

for g in train_data_list:
      train_folder_path                                                                    =
"/home/behavior/Desktop/python/LearnFace/3_TrainFaceImage/6to8/" + g
      for i in layer_list:
            for j in learning_rate_list:

                        d_today = datetime.datetime.now()
                        weight_number = d_today.strftime('%m%d%H%M')

                        #Path setting

                        result_folder_path                                         =
```

```
'/home/behavior/Desktop/python/LearnFace/4_FaceTest'+ '/Result_' + weight_number
                        test_img_folder_path                                                                  =
'/home/behavior/Desktop/python/LearnFace/3_TrainFaceImage/6to8/original/test_data'
                        weight_path  =  result_folder_path  +  '/Marmoset_face_recognition_'  +
weight_number +'_vgg19.hdf5'

                        #Result directory
                        os.makedirs(result_folder_path, exist_ok=True)

                        train_face(i, j)
                        test_face()
                        result_csv()

print('****learn_face is done.****')
```
