# Supplementary material for: Development of a 3D tracking system for multiple marmosets under free-moving conditions
Source: Commun Biol. 2024 Feb 21;7:216. doi: 10.1038/s42003-024-05864-9 (PMC10881507; doi:10.1038/s42003-024-05864-9)
Supplement: Supplementary file 13 — Supplementary Data 2 [file 42003_2024_5864_MOESM13_ESM.pdf]

Code for grooming detection

```
/**
  * Filter by Gaussian weighted moving average over a fixed time slide and window width for a fixed time.¥n
  * Gaussian weighted moving average processing is performed for each behavior type, and only the behavior data in the time zone exceeding the threshold value is extracted.
  * @brief Gaussian weighted moving average filter function
  * @param all_behavior_map Behavior detection result information detected by all cameras
  * @param FILTER_THRESHOLD Filter threshold
  * @param FILTER_GRID_TIME Data subdivision time for filters (unit: seconds)
  * @param FILTER_SLIDE_TIME Time to slide the filter range (unit: seconds)
  * @param FILTER_WINDOW_TIME Filter window time width (unit: seconds)
  * @param file_start_unixtime Video file start time
  * @param file_end_unixtime Video file end time
  * @return std::map<double, std::vector<behavior_info_csv_divide_writer::BehaviorInfoCsvDivideWriter::Behavior>> Behavior detection result information detected by all cameras after filtering
  */
std::map<double,
std::vector<behavior_info_csv_divide_writer::BehaviorInfoCsvDivideWriter::Behavior>>
behavior_info_csv_divide_writer::BehaviorInfoCsvDivideWriter::gaussianWeightedMovingAverage(std::map<double,
std::vector<behavior_info_csv_divide_writer::BehaviorInfoCsvDivideWriter::Behavior>>
all_behavior_map, double FILTER_THRESHOLD, double FILTER_GRID_TIME, double FILTER_SLIDE_TIME, double FILTER_WINDOW_TIME, double file_start_unixtime, double file_end_unixtime){
	std::map<double,
std::vector<behavior_info_csv_divide_writer::BehaviorInfoCsvDivideWriter::Behavior>>
result_all_behavior_filter_map;
	// Divide the number of behavior detections into a time range (grid) at regular intervals for each behavior type.
	std::map<std::string,
std::vector<behavior_info_csv_divide_writer::BehaviorInfoCsvDivideWriter::Grid>>
tmp_grid_per_behavior_map = makeGrid(all_behavior_map, FILTER_GRID_TIME, file_start_unixtime, file_end_unixtime);
```

```
        // Add the acquired grid data
        for(auto& itr : tmp_grid_per_behavior_map){

        grid_per_behavior_map[itr.first].insert(grid_per_behavior_map[itr.first].end(), itr.second.begin(), itr.second.end());
        }
        // Pass the grid for each behavior type through a Gaussian weighted moving average filter
        unsigned int residue_grid_size = 0;
        for(auto& behavior_itr : grid_per_behavior_map){
                unsigned int slide_grid_size = std::round(FILTER_SLIDE_TIME / FILTER_GRID_TIME);
                unsigned int window_grid_size = std::round(FILTER_WINDOW_TIME / FILTER_GRID_TIME);
                unsigned int window_grid_range = window_grid_size;
                unsigned int grid_max = behavior_itr.second.size();
                double calc_completed_grid_unixtime = 0.0;
                for(unsigned int grid_num = 0; grid_num < behavior_itr.second.size(); grid_num++){
                        // Slide the filter calculation range for a certain period of time
                        if(grid_num % slide_grid_size == 0){
                                window_grid_range = grid_num + window_grid_size;
                                // If the end time of the next X second slide & XX second window exceeds the end time of one video file, the filtering process is interrupted.
                                if(grid_max <= window_grid_range) break;
                                // Calculate Gaussian weighted moving average in window range
                                bool block_f = false;
                                double old_grid_unixtime = behavior_itr.second[grid_num].grid_unixtime;
                                double ysum = 0.0;
                                for(unsigned int calc_window_grid_num = grid_num; calc_window_grid_num < window_grid_range; calc_window_grid_num++){
                                        double diff_grid_unixtime = behavior_itr.second[calc_window_grid_num].grid_unixtime - old_grid_unixtime;
                                        if((FILTER_WINDOW_TIME <
```

```
diff_grid_unixtime) && block_f == false){
                        if(old_residue_grid_size == 0) break;
                        window_grid_range = old_residue_grid_size - 1;
                        block_f = true;
                    }
                    old_grid_unixtime = behavior_itr.second[calc_window_grid_num].grid_unixtime;
                    // Gaussian weighted moving average calculation
                    double x = ((grid_num + FILTER_WINDOW_TIME) - calc_window_grid_num) / FILTER_WINDOW_TIME * 3.0;
                    double y = std::exp(-1 * std::pow(x, 2) / 2) / std::sqrt(2 * M_PI); // Standard normal distribution
                    ysum = ysum + y * behavior_itr.second[calc_window_grid_num].behavior_count;

                }
                unsigned int next_slide_grid_num = grid_num + slide_grid_size;
                if(grid_max <= next_slide_grid_num) next_slide_grid_num = grid_max -1;
                // Save the behavior when the filter value exceeds the threshold
                if(FILTER_THRESHOLD <= ysum){
                    for(unsigned int get_grid_data_num = grid_num; get_grid_data_num < next_slide_grid_num; get_grid_data_num++){
                        for(auto& data_itr : behavior_itr.second[get_grid_data_num].behavior_map){

    result_all_behavior_filter_map[data_itr.first].insert(result_all_behavior_filter_map[data_itr.first].end(), data_itr.second.begin(), data_itr.second.end());
                        }
                    }
                }
                calc_completed_grid_unixtime =
```

```
behavior_itr.second[next_slide_grid_num - 1].grid_unixtime;
                    }
                }
                // Delete unnecessary grid data
                for(int clear_grid_num = behavior_itr.second.size() - 1; 0 <=
clear_grid_num; clear_grid_num--){
                    if( behavior_itr.second[clear_grid_num].grid_unixtime <=
calc_completed_grid_unixtime ){
                        behavior_itr.second.erase(behavior_itr.second.begin()
+ clear_grid_num);
                    }
                }
                residue_grid_size = behavior_itr.second.size();
        }
        old_residue_grid_size = residue_grid_size;
        return result_all_behavior_filter_map;
}
```
