# Supplementary material for: Development of a 3D tracking system for multiple marmosets under free-moving conditions
Source: Commun Biol. 2024 Feb 21;7:216. doi: 10.1038/s42003-024-05864-9 (PMC10881507; doi:10.1038/s42003-024-05864-9)
Supplement: Supplementary file 15 — Reporting Summary [file 42003_2024_5864_MOESM15_ESM.pdf]

nature portfolio

Corresponding author(s): Erika Sasaki

Last updated by author(s): Jan 19, 2024

Reporting Summary

Nature Portfolio wishes to improve the reproducibility of the work that we publish. This form provides structure for consistency and transparency in reporting. For further information on Nature Portfolio policies, see our Editorial Policies and the Editorial Policy Checklist.

Statistics

For all statistical analyses, confirm that the following items are present in the figure legend, table legend, main text, or Methods section.

n/a	Confirmed	
☐	☒	The exact sample size (*n*) for each experimental group/condition, given as a discrete number and unit of measurement
☐	☒	A statement on whether measurements were taken from distinct samples or whether the same sample was measured repeatedly
☐	☒	The statistical test(s) used AND whether they are one- or two-sided<br>*Only common tests should be described solely by name; describe more complex techniques in the Methods section.*
☒	☐	A description of all covariates tested
☒	☐	A description of any assumptions or corrections, such as tests of normality and adjustment for multiple comparisons
☐	☒	A full description of the statistical parameters including central tendency (e.g. means) or other basic estimates (e.g. regression coefficient) AND variation (e.g. standard deviation) or associated estimates of uncertainty (e.g. confidence intervals)
☒	☐	For null hypothesis testing, the test statistic (e.g. *F*, *t*, *r*) with confidence intervals, effect sizes, degrees of freedom and *P* value noted<br>*Give P values as exact values whenever suitable.*
☒	☐	For Bayesian analysis, information on the choice of priors and Markov chain Monte Carlo settings
☒	☐	For hierarchical and complex designs, identification of the appropriate level for tests and full reporting of outcomes
☒	☐	Estimates of effect sizes (e.g. Cohen's *d*, Pearson's *r*), indicating how they were calculated

Our web collection on statistics for biologists contains articles on many of the points above.

Software and code

Policy information about availability of computer code

Data collection	Lidar (VLP-16, Velodyne Lidar) and camera (C920, Logitech) were used to collect marmoset data. Software was developed by Hitachi, Ltd.
Data analysis	The analysis was carried out using a self-made machine learning programme, the open-source Yolo v3 and a programme developed by Hitachi, Ltd.

For manuscripts utilizing custom algorithms or software that are central to the research but not yet described in published literature, software must be made available to editors and reviewers. We strongly encourage code deposition in a community repository (e.g. GitHub). See the Nature Portfolio guidelines for submitting code & software for further information.

Data

Policy information about availability of data

All manuscripts must include a data availability statement. This statement should provide the following information, where applicable:

- Accession codes, unique identifiers, or web links for publicly available datasets
- A description of any restrictions on data availability
- For clinical datasets or third party data, please ensure that the statement adheres to our policy

The readily tested code and data has been presented in the Supplemental information (Supplementary Data S1, S2). Information and requests for the program for linkage between video tracking and Lidar should be directed to and will be fulfilled by Norio Goda (norio.goda.ws@hitachi.com).

Human research participants

Policy information about studies involving human research participants and Sex and Gender in Research.

Reporting on sex and gender	The study did not involve human.
Population characteristics	The study did not involve human.
Recruitment	The study did not involve human.
Ethics oversight	The study did not involve human.

Note that full information on the approval of the study protocol must also be provided in the manuscript.

Field-specific reporting

Please select the one below that is the best fit for your research. If you are not sure, read the appropriate sections before making your selection.

☒ Life sciences ☐ Behavioural & social sciences ☐ Ecological, evolutionary & environmental sciences

For a reference copy of the document with all sections, see nature.com/documents/nr-reporting-summary-flat.pdf

Life sciences study design

All studies must disclose on these points even when the disclosure is negative.

Sample size	The number of frames was prepared up to the number of images for which the learning accuracy was sufficient, without pre-determining the number of samples. No statistical methods were used for predetermining sample sizes. The number of animals used were minimized.
Data exclusions	No data was excluded.
Replication	Face identification was verified with several trainings and animals, and similar results were obtained in all trainings. Tracking was validated with multiple animals within the angle of view, with similar accuracy in all animals compared to visual inspection.
Randomization	Data was randomly assigned to the training or test splits.
Blinding	Labelers were not told which videos or labeled data would be included in the training or test splits.

Reporting for specific materials, systems and methods

We require information from authors about some types of materials, experimental systems and methods used in many studies. Here, indicate whether each material, system or method listed is relevant to your study. If you are not sure if a list item applies to your research, read the appropriate section before selecting a response.

Materials & experimental systems

n/a	Involved in the study	
☒	☐	Antibodies
☒	☐	Eukaryotic cell lines
☒	☐	Palaeontology and archaeology
☐	☒	Animals and other organisms
☒	☐	Clinical data
☒	☐	Dual use research of concern

Methods

n/a	Involved in the study	
☒	☐	ChIP-seq
☒	☐	Flow cytometry
☒	☐	MRI-based neuroimaging

Animals and other research organisms

Policy information about studies involving animals; ARRIVE guidelines recommended for reporting animal research, and Sex and Gender in Research

Laboratory animals	Conventional common marmosets (1-10 years, male and female) purchased from CLEA Japan, Inc. (Tokyo, Japan) or bred in CIEA were

	used.
Wild animals	The study did not involve wild animals.
Reporting on sex	This study involved both sexes.
Field-collected samples	No field-collected samples were used.
Ethics oversight	This study was conducted at the animal facility of the Central Institute for Experimental Animals (CIEA), Kawasaki, Japan. The animal experimental protocol was reviewed by the Institutional Animal Care and Use Committee and approved (approval no. 17051) according to the Regulations for Animal Experiments in CIEA, based on the Guidelines for the Proper Conduct of Animal Experiments by the Science Council of Japan (2006).

Note that full information on the approval of the study protocol must also be provided in the manuscript.
